# Editorial: Enzyme or Whole Cell Immobilization for Efficient Biocatalysis: Focusing on Novel Supporting Platforms and Immobilization Techniques

**DOI:** 10.3389/fbioe.2021.620292

**Published:** 2021-03-01

**Authors:** Wen-Yong Lou, Jesús Fernández-Lucas, Jun Ge, Changzhu Wu

**Affiliations:** ^1^Laboratory of Applied Biocatalysis, School of Food Science and Engineering, South China University of Technology, Guangzhou, China; ^2^Applied Biotechnology Group, Universidad Europea de Madrid, Urbanización El Bosque, Madrid, Spain; ^3^Grupo de Investigación en Ciencias Naturales y Exactas, GICNEX, Universidad de la Costa, CUC, Barranquilla, Colombia; ^4^Key Laboratory of Industrial Biocatalysis, Ministry of Education, Department of Chemical Engineering, Tsinghua University, Beijing, China; ^5^Department of Physics, Chemistry and Pharmacy University of Southern Denmark, Odense, Denmark

**Keywords:** enzyme immobilization, whole-cell immobilization, biotransformation, biocatalysis, immobilization materials

Biocatalysts represented by enzymes and enzyme-containing whole cells are generally fragile and easily inactivated in practical application conditions. The immobilization concept and techniques have been recognized as classic and powerful strategy for tackling such challenges (Hanefeld et al., 2009). Based on this background, a special Research Topic entitled *Enzyme or Whole Cell Immobilization for Efficient Biocatalysis: Focusing on Novel Supporting Platforms and Immobilization Techniques* had been organized and presented in the platform of *Frontiers in Bioengineering and Biotechnology*, which aimed to collect different insights and latest findings regarding but not limited to new theories, techniques and methodologies in this area. Over the past year since Sept. 2019, this Research Topic has attracted 242 authors from more than 10 countries to participate and contribute their manuscripts. Consequently, this special issue has selected and presented 40 peer-reviewed articles to meet the readers, including 31 Original Researches, four Brief Research Reports, four Reviews, and one General Commentary, which involved various aspects and every corner of this area.

When it refers to the concept of immobilization, a crucial thing that foremost comes to mind is the supporting platform, which belongs to the most concerned issues of this Research Topic. The design or discovery of novel enzyme/whole cell supporting materials with desired properties and performances (e.g., high biocatalysts loading efficiency, favorable chemical and mechanical stability, wide applicability, high biocompatibility, as well as low cost) are fairly important. In this context, multifarious immobilization carriers have been reported in this Research Topic, ranging from natural origin matrixes to artificial synthetic materials (e.g., porous, polymeric, nanostructured or magnetic materials) and more. From the aspect of natural origin matrixes, for instance, Lin et al. employed the deep eutectic solvents-treated chitosan as supporting platform for immobilizing papain, realizing enhanced thermostability of the guest enzyme, thanks to the microporous structure and suitable catalytic microenvironment of the immobilized enzyme. Other natural matrixes or their derivatives, including agarose spheres or beads (Li, Zhang et al., Ahumada et al.), pichia pastoris (Han et al.), glyoxyl- and octyl-agarose (Ubilla et al., Arana-Peña et al.), novel affinity tag ChBD-AB from *Chitinolyticbacter meiyuanensis* SYBC-H1 (Zhou, Chen et al.) and chitin (Zhou, Zhang et al.), were also well designed and employed for immobilization of various enzymes, demonstrating their green, sustainable, and high biocompatibility properties.

Synthetic materials are very intricate but prevalent and versatile supporting matrixes that have been widely employed. These materials can be empirically divided into inorganic carriers, cellular or mesoporous materials, polymeric materials, microcapsules, nanomaterials and magnetic materials, though they may show certain overlap in the taxonomy. Specifically, for instance, Wang, Owusu-Fordjour et al. developed various metal-chelated magnetic silica nanoparticles for immobilizing laccase, which were further used for apple juice clarification. Other representative and interesting artificial synthetic materials, such as metal ions modified hollow mesoporous silica spheres (HMSS-NH_2_-Metal, Zeng et al.), mesostructured cellular foams (Kumpf et al.), Fe_3_O_4_-based functional materials (Fe_3_O_4_@graphene oxide, Wan et al.; magnetic Fe_3_O_4_@polydopamine, Bi et al.; Fe_3_O_4_@chitosan, Liu et al.; Fe_3_O_4_@3-aminopropyltriethoxysilane, Moreira et al.), metal-organic frameworks (MOFs, Lei et al., Xia et al., Li et al., Wang et al., Xu et al.), mesoporous CaCO_3_ microspheres (Lee et al., Han et al.) and traditional resin materials (Guo et al., Yang et al.), were also rationally fabricated and employed, showing huge potential in improving the catalytic performances of the immobilized biocatalysts. Among them, it is worth noting that MOFs have emerged as attritive and efficient supporting matrixes for enzyme or whole cell immobilization, attribute to their unique features such as high specific surface area and pore volumes, tunable and uniform topological structure, as well as competent stability in certain environments (Liang et al., 2020). For instance, Wang, Zhang et al. developed a facile method for synthesis of *Candida antarctica* lipase-embedded MOFs (CalB@ZIF-8), which was successfully applied in size-selective transesterification reaction with higher catalytic activity and thermostability, due to the intrinsic micropores of the robust ZIF-8 shell.

In addition to abovementioned materials, some commercially available enzyme preparations in the immobilized forms were also employed, mainly focusing on their practical applications (Xia, Wan et al., Chen et al., Ma et al.). These fully-developed enzyme preparations are not only the beacons for the development of immobilized biocatalysts, but also the goals we want to approach or even surpass. Moreover, carrier-free (Zang et al.), hybrid nanoflower (Wu et al.) and functional nanomembrane (Mazzei et al.) for enzyme or whole cell immobilization were also recorded in this Research Topic. The review articles, presented by Rodriguez-Abetxuko et al., Xu et al., and Xia, Li et al., systematically introduced various kinds of supporting scaffolds for biocatalysts immobilization, which provide more information for the readers with further interests.

The advanced techniques for enzyme or whole cell immobilization and other relevant frontier methodologies are also the major concerns of this Research Topic. Generally, enzyme or whole cell can be immobilized on/in the selected supports via principal routes of adsorption, covalent binding, cross-linking or encapsulation (Taheri-Kafrani et al., 2000). Following the general principles, nevertheless, a large number of cutting-edge technologies emerged in our Research Topic articles. For instance, Han et al. constructed a novel cell surface display system for anchoring *Thermomyces lanuginosus* lipase (TLL) on the yeast cell wall, realizing a robust TLL whole cell biocatalyst with higher thermostability and alkaline pH resistance. In another report, Zhou, Chen et al. constructed a facile and efficient protein purification and immobilization system with novel ChBD-AB affinity tag, which exhibited advantages such as cost-saving, environmental-friendly, simple operation, rapidness, and high-purity. Similarly, a one-step programme for purification and immobilization of lysine decarboxylase (CadA) via fusion of a chitin-binding domain (ChBD) was established by Zhou, Zhang et al., which was further used for production of cadaverine from L-lysine. Surface modification of poly glycidyl methacrylate (PGMA) spheres via a combination of plasma and amination for multienzyme immobilization and cascade catalysis was reported by Liao et al., which is a representative case in the attempting of the cutting-edge technologies and concepts. Analogously, a layer-by-layer self-assembly strategy, raised by Li, Wen et al., for sequential co-immobilization of multienzyme within MOFs for further transformation of CO_2_, was also commendable. In terms of analytical methods, Yan et al. successfully developed a sensitive and reproducible SERS spectra technique for single bacterial cell analysis. Moreover, the bioreactors based on the immobilized enzymes, including the integrated biomembrane (Mazzei et al.), a packed bed reactor (Li, Zhang et al.), pickering emulsion-based microreactors (Lei et al.), magnetically stabilized fluidized bed (Wang, Owusu-Fordjour et al.), bubble column reactor (Chen et al.), and the biofiltration system packed with fly ash ceramsite (Sun et al.), are also the technological highlights given that they can greatly improve the catalytic performances. It should be emphasized that the abovementioned techniques, methodologies or concepts are probably not the pioneering works, but they do belong to the technical frontiers of this field, which will greatly promote the robust development of this area.

Beyond the supporting platforms and immobilization techniques, the catalytic process regulation of the immobilized biocatalysts is another attractive and crucial topic of this special issue. Solvent engineering-based regulating strategies, especially the biocatalysis in non-aqueous systems, have attracted substantial attentions. Deep eutectic solvents (DESs) are representative and prevailing non-aqueous mediums for regulation and optimization of biocatalytic reactions, which can be designed as non-toxic, non-volatile, non-flammable, or even biodegradable liquids for improving the substrate supply, conversion, and stability (Pätzold et al., 2019). In an Original Research article, Zang et al. employed the natural DESs as hydrolysis solvents for the carrier-free enzyme aggregate of rutin degrading enzyme, proving that DESs are valuable for improving enzyme activity and stability in practical conditions. The pre-treatment of the supporting materials, such as chitosan (Lin et al.) and chitin nanocrystal (Jiang et al.), by DESs also play a significant role for improving the performances of immobilized biocatalysts.

Organic solvents-based non-aqueous systems are also very important mediums for biotransformation reactions as they can modulates biocatalyst activity, selectivity, and stability. As a representative case, Yang, Nie et al. developed a cosolvent system containing tetrahydrofuran and isopropyl ether for enhancing the biosynthesis of aromatic esters of arbutin by immobilized lipase from *Penicillium expansum*. Results showed that the initial rate and substrate conversion of arbutin vanilylation were markedly enhanced as compared to the single tetrahydrofuran solvent. Similarly, a ternary cosolvent system (acetone/1-butanol/aqueous solution) was developed by Ahumada et al. for regulating the synthesis of butyl-β-galactoside using soluble or immobilized *Aspergillus oryzae* β-galactosidase, which is expected to favor the transgalactosylation reaction. Moreover, the organic solvents mediated regulation of the biocatalytic process of other lipases (Chen et al., Wang, Zhang et al., and Bi et al.) were also reported in our Research Topic. In addition to DESs and organic solvents, pickering emulsion-based medium for size-selective enzymatic catalysis of transesterification reaction by MOFs-immobilized lipase is another interesting case (Lei et al.). Beyond abovementioned strategies based on solvent engineering, a commonplace but indeed very effective method is the optimization of process parameters, which were also widely reported in this Research Topic (Arana-Peña et al., Peng et al., Chen et al., etc.).

Insights into the interaction mechanisms between enzyme/whole cell and the supporting platforms would benefit the in-depth rational design of efficient biocatalytic systems. In this respect, for example, Lee et al. employed a variety of modern instrument analytical techniques for characterization of the supporting platform (CaCO_3_ microspheres) and immobilized enzyme (carboxyl esterase), proving that the enzyme was entrapped and then cross-linked to form a stable enzyme@CaCO_3_ microsphere structure. In the case of the alcalase@HMSS-NH_2_-Metal, the authors demonstrated that alcalase was immobilized on the tailor-made platforms via a metal-protein affinity, where the binding interaction between metal ions and enzyme could promote the transformation of the enzyme secondary structure, resulting in the significantly improvement of catalytic activity and thermostability of alcalase (Zeng et al.). Another noteworthy article focusing on the mechanism explanation was reported by Chen et al., which investigated the conformational stability of porcine pancreatic lipase (PPL) in various non-aqueous solvents by using Molecular Dynamic (MD) simulation, indicating that DMSO was the prefer solvent given that the enzyme retained a loose and extended native backbone in this solvent. This study demonstrated the robust development and huge potential of the computational chemistry-based theories and methodologies for guiding the development of the biocatalysis area.

Multienzyme cascade and immobilization for biocatalysis, which is an emerging and hot concept in this area, has captured a lot of attentions from the contributors. In the multienzyme cascade, the reactions occur sequentially, in which the product of the previous reaction is used as the substrate of the next reaction (Ren et al., 2019). It is usually very difficult to design an ideal multienzyme cascade system because both the substrates and biocatalysts must be effectively activated, while the co-immobilization of multienzyme within suitable platform provides an alternative route for construction of efficient biocatalysts for multienzyme-involved reactions. In a representative Original Research article, Li, Wen et al. investigated the sequential co-immobilization of carbonic anhydrase, formate dehydrogenase and glutamate dehydrogenase within core-shell structured metal-organic works for adsorption and biotransformation of CO_2_ to formate, which exhibited a 13.1-times higher product yield attribute to the enrichment of substrate by the special layer-by-layer multienzyme system. In another case, Han et al. performed the co-immobilization of three enzymes on porous CaCO_3_ microspheres for production of inositol from starch, which displayed improved catalytic activity (~3.5 fold) than the individual immobilized enzymes, as well as enhanced thermostability and reusability than free enzyme mixture. The co-immobilization of glucose oxidase and catalase within modified PGMA spheres was also reported, showing extensive range of temperature and pH adaptability (Liao et al.).

In addition to multienzyme immobilization, Peng et al. explored the coexpression of (*2R, 3R*)-butanediol dehydrogenase (*Kg*BDH) and glucose dehydrogenase (GDH) in recombinant *Escherichia coli* strain, followed by immobilization of the whole cell using a mixture of activated carbon and calcium alginate, realizing a cofactor self-sufficient whole cell biocatalyst for enantioselective synthesis of (*R*)-1-phenyl-1,2-ethanediol with product yield of 81% and >99% enantiomeric excess (*ee*) value. Moreover, beyond the Original Research articles, the frontiers of multienzyme immobilization and cascade were also well-summarized in the review articles. For example, Martínez-Rodríguez et al. presented a comprehensive review about the developments and advances of multienzymatic cascades for production of non-canonical α-amino acids. More specifically, Xu et al. summarized the progress of materials-based techniques for multienzyme immobilization from both aspects of the immobilization strategies and supporting materials. In addition, MOFs as novel and versatile supporting matrixes for multienzyme immobilization was also highlighted in the review of Xu et al. Despite the development of multienzyme immobilization and cascade is still in its infancy, these cases demonstrated the popularity and boundless potentiality of this emerging concept.

On the foundation of abovementioned items, the ultimate purpose of enzyme or whole cell immobilization is designed for the practical application, which involves a wide range of aspects. In this present Research Topic, the applications of immobilized enzymes or whole cells can be summarized as follows: (1) the biosynthesis of high value-added chemicals; (2) the elimination of environmental hazardous substances; and (3) the processing of food materials for the quality or safety purposes. For the purpose of high value-added chemicals preparations, e.g., Xia, Wan et al. successfully synthesized a hydrophobic propionyl neohesperidin ester (PNHE) by modification of neohesperidin using immobilized lipase, the resulting PNHE was used for antiproliferation and pro-apoptosis of human MCF-7 breast cancer cells with improved activity as compared to neohesperidin. Kumpf et al. investigated the identification, gene expression, purification and immobilization of uridine-5'-diphosphate-glucose (UDP-glucose) pyrophosphorylase for production of UDP-glucose, which is a fundamentally important molecule in biology, food, biopharmaceuticals as well as cosmetic chemistry. Biosynthesizes of other high-value added chemicals were also extensively reported in this Research Topic, including oleuropein aglycone (Mazzei et al.), butyl-β-D-galactoside (Ahumada et al.), lactulose (Ubilla et al.), nucleoside analogs (Acosta et al.), hesperetin-7-*O*-glucoside (HMG, Wan et al.), medium chain diacylglycerol (MCD, Chen et al.), butyric acid (Liu et al.), inositol (Han et al.), aromatic esters of arbutin (Yang, Nie et al.), cadaverine (Zhou, Zhang et al.), (*R*)-1-phenyl-1,2-ethanediol (Peng et al.), and biodiesel (Ma et al.). It is worth noting that the synthesizes of nucleoside analogs reported by Acosta et al.ss belong to the upstream edge of this Research Topic, which emphasized the developments of new enzymes by genetic engineering technology.

For the applications in the environmental aspect, a typical case is the transformation of CO_2_ to formate by using MOFs-based multienzyme immobilization system (Li, Wen et al.), providing an alternative and effective strategy for alleviating the CO_2_ crisis. In another interesting work, Sun et al. developed a new methane elimination system by using biofiltration packed with fly ash ceramsite (FAC). The use of FAC supporting material in methane biofiltration system would allow the industrial and scaled application of methane-oxidizing bacteria in methane elimination (Sun et al.). The production of biodiesel by using immobilized lipase, reported by Ma et al. can be also classified as an application in the environmental aspect.

In the food-related aspect, Li, Zhang et al. developed a packed bed reactor of immobilized L-asparaginase for reducing the formation of harmful acrylamide in the fluid food model system, which is useful from the perspective of food safety. Wang, Owusu-Fordjour et al. investigated the immobilizations of laccase on magnetic chelator nanoparticles and their applications in apple juice clarification, resulting in higher freeze-thaw and thermal stability of the apple juice. Moreover, the concentration of DHA (docosahexaenoic acid) and EPA (eicosapentaenoic acid) in glycerides by immobilized lipase was reported by Ma et al., which is another representative case of the food-related application. The abovementioned cases demonstrated the practicalities of the immobilized biocatalysts, however, it is a little pity that the applications of them in the fields of biosensing and disease diagnosis & therapy were not included in this Research Topic.

In summary, this Research Topic presents a comprehensive exhibition of the general knowledge and representative frontier developments about the field of enzyme or whole cell immobilization. The crucial aspects of this field, including the supporting platforms, immobilization or other relevant techniques, biocatalytic process regulations, mechanism interpretations, as well as the practical applications, are comprehensively included in the collected articles. This Research Topic provides an ideal platform for the peer experts to exhibit their latest achievements, and we hope that the anthology will provide useful references and inspirations to the peer readers. On the other hand, we should also recognize the deficiencies and limitations of this Research Topic, such as lack of phenomenal findings and imbalance in geographical distribution of contributors. Although there presents several intractably bottlenecks in this field, with the joint efforts of the researchers, we firmly believe that the enzyme or whole cell immobilization field will make remarkable achievements in the future.

We would like to thank all the authors, reviewers, and the Editorial Board members for their considerable contributions to support the implementation of this special Research Topic. We also sincerely appreciate Mr. Shan Liang for his selfless dedication throughout the whole process in proceeding the Research Topic.

## Author Contributions

W-YL drafted the Editorial. All authors listed have made a substantial, direct and intellectual contribution to the work, and approved it for publication.

## Conflict of Interest

The authors declare that the research was conducted in the absence of any commercial or financial relationships that could be construed as a potential conflict of interest.
